# Effect of Yttrium Oxide on Microstructure and Oxidation Behavior of Cr/FeCrAl Coatings Fabricated by Extreme High-Speed Laser Cladding Process: An Experimental Approach

**DOI:** 10.3390/ma18081821

**Published:** 2025-04-16

**Authors:** Tian Liang, Jian Liu, Chi Zhan, Shaoyuan Peng, Jibin Pu

**Affiliations:** 1 School of Materials Science and Chemical Engineering, Ningbo University, Ningbo 315211, China; liangtian@nimte.ac.cn; 2 State Key Laboratory of Advanced Marine Materials, Ningbo Institute of Materials Technology and Engineering, Chinese Academy of Sciences, Ningbo 315201, China; liujian123@nimte.ac.cn; 3 China Merchants Marine and Offshore Research Institute Co., Ltd., Shenzhen 518066, China; zhanchi1@cmhk.com

**Keywords:** extreme high-speed laser cladding, FeCrAl coating, rare earth oxide, Zr-4 alloy, microstructure, high-temperature steam oxidation, loss of coolant accident

## Abstract

Zr-4 alloy tubes, as the primary cladding material in nuclear reactor cores, face the critical challenge of oxidative attack in 1200 °C steam environments. To address this issue, high-temperature oxidation-resistant coatings fabricated via extreme high-speed laser cladding (EHLA) present a promising mitigation strategy. In this study, Y_2_O_3_-modified (0.0–5.0 wt.%) Cr/FeCrAl composite coatings were designed and fabricated on Zr-4 substrates using the EHLA process, followed by systematic investigation of Y doping effects on coating microstructures and steam oxidation resistance (1200 °C, H_2_O atmosphere). Experimental results demonstrate that Y_2_O_3_ doping remarkably enhanced the oxidation resistance, with optimal performance achieved at 2.0 wt.% Y_2_O_3_ (31% oxidation mass gain compared to the substrate after 120-min exposure). Microstructural analysis reveals that the dense grain boundary network facilitates rapid surface diffusion of Al, promoting continuous Al_2_O_3_ protective film formation. Additionally, Y segregation at grain boundaries suppressed outward diffusion of Cr^3+^ cations, effectively inhibiting void formation at the oxide-coating interface and improving interfacial stability. The developed rare-earth-oxide-doped composite coating via extreme high-speed laser cladding process shows promising applications in surface-strengthening engineering for nuclear reactor Zr-4 alloy cladding tubes, providing both theoretical insights and technical references for the design of high-temperature oxidation-resistant coatings in nuclear industry.

## 1. Introduction

Nuclear energy, as a clean and efficient energy source, plays a crucial role in optimizing the global energy structure and ensuring energy supply [1]. Zirconium alloys, characterized by superior corrosion resistance, mechanical plasticity, and neutron economy, remain the exclusive cladding materials for water-cooled nuclear reactors [2]. However, a loss of coolant accident (LOCA) in nuclear reactors can lead to a rapid increase in core temperature (up to 1200 °C), causing a violent reaction between zirconium alloy cladding and high-temperature steam. This reaction generates a large amount of hydrogen and heat, forming a detrimental “hydrogen explosion–temperature rise” vicious cycle. To enhance LOCA tolerance, surface coating modifications to the existing zirconium alloy cladding are an effective solution to improve the cladding’s resistance to extreme high-temperature oxidation while retaining the advantageous properties of zirconium alloys [3].

The deposition of metal coatings such as FeCrAl and Cr on the surface of zirconium alloy has been demonstrated as an effective approach to enhance its resistance to high-temperature oxidation [4,5]. Among these, FeCrAl coatings have garnered significant attention due to their operational response characteristics, generating a protective layer primarily composed of Cr_2_O_3_ under normal operating conditions [6] and a stable high-temperature Al_2_O_3_ oxide film under accident conditions [7]. Various coating preparation techniques have been reported for the fabrication of protective coatings on zirconium alloys, including magnetron sputtering [8], thermal/cold spraying [9,10], and laser cladding [11]. Compared to other coating preparation methods, extreme high-speed laser cladding has demonstrated superior technical advantages for zirconium alloy coatings, including enhanced processing velocity, reduced dilution rates, minimized thermal distortion, and improved deposition efficiency [12]. Nevertheless, significant Fe-Zr interdiffusion occurs at 928 °C, forming a low-melting-point eutectic that significantly accelerates the coating degradation rate, thereby greatly reducing its oxidation resistance. Furthermore, investigations of interfacial evolution and elemental migration in low-aluminum FeCrAl coatings indicate that the outward migration of Al and the resulting pores, along with the peeling of the protective Al_2_O_3_ film, are additional primary factors contributing to the degradation of FeCrAl coatings [13]. These issues make it difficult for existing FeCrAl coatings to effectively protect zirconium alloy cladding from extreme high-temperature oxidation damage at 1200 °C.

Recent studies propose that the deposition of a Cr intermediate layer between the FeCrAl coating and the zirconium alloy substrate can effectively delay the rapid Fe-Zr interdiffusion, thereby improving the coating’s oxidation resistance in a 1200 °C steam environment [14,15]. On the other hand, the doping of rare earth element Y can alter the oxidation layer growth mechanism, suppress the formation of voids and defects at the Al_2_O_3_-coating interface due to diffusion, and enhance the adhesion of the oxide layer on the coating surface [16]. For instance, yttrium-modified FeCrAl coatings demonstrate improved Al_2_O_3_ film formation capability in oxygen-enriched lead-bismuth eutectic environments (106 wt% O_2_) compared to unmodified counterparts [17]. However, the specific effects and mechanisms of rare earth Y on FeCrAl coating microstructure and high-temperature steam oxidation behavior remain insufficiently understood. Therefore, this study systematically investigates the influence of Y_2_O_3_ on the microstructure and high-temperature oxidation resistance (1200 °C steam environment) of extreme high-speed laser-clad Cr/FeCrAl composite coatings. The intrinsic correlations between the composition, microstructure, and high-temperature oxidation behavior of the coatings are established, and the mechanism behind the enhancement of the coatings’ oxidation resistance is thoroughly revealed. The findings of this research are expected to provide theoretical guidance and technical references for the design and preparation of high-tolerance protective coatings for nuclear reactors in the event of a loss of coolant accident.

## 2. Experimental Approaches

### 2.1. Materials

**Figure 1:**
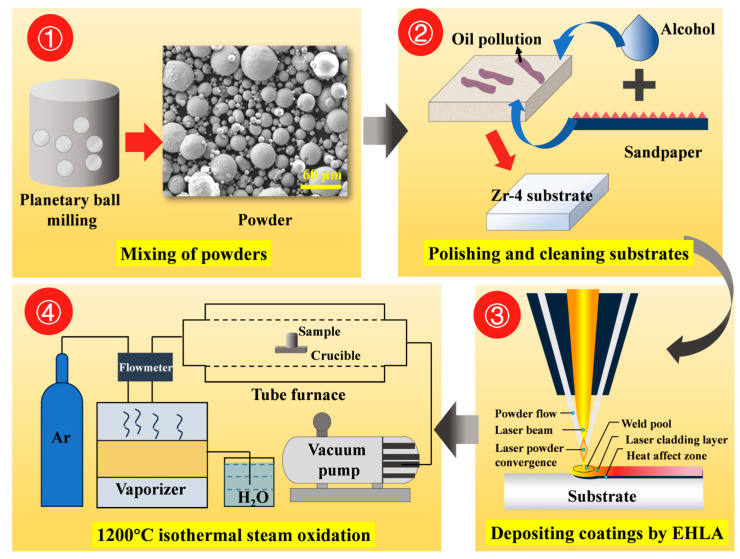
Experimental methodology flowchart.

Commercially used Zr-4 alloy tubing (supplied by Western Xincai Technology Co., Ltd., Xi’an, China) was employed as the substrate in this study, with its chemical composition provided in Table 1. The tubing dimensions were 9.5-mm outer diameter, 0.57-mm wall thickness, and 300-mm length. Spherical FeCrAl alloy powder (commercial grade) and pure chromium powder (99.99% purity, particle size: 30–70 μm, supplied by Nanchang Guocai Technology Co., Ltd., Nanchang, China) served as coating materials. Yttrium oxide (Y_2_O_3_) particles (99.99% purity, particle size: 0.5 μm, sourced from Shanghai Maclin Biochemical Technology Co., Ltd., Shanghai, China) were blended with FeCrAl alloy powder at varying mass ratios using planetary ball milling. Table 2 presents the elemental composition of Cr/FeCrAl-based coatings with different Y_2_O_3_ additions. Figure 1 shows the experimental methodology flowchart.

### 2.2. Coating Preparation

**Figure 2:**
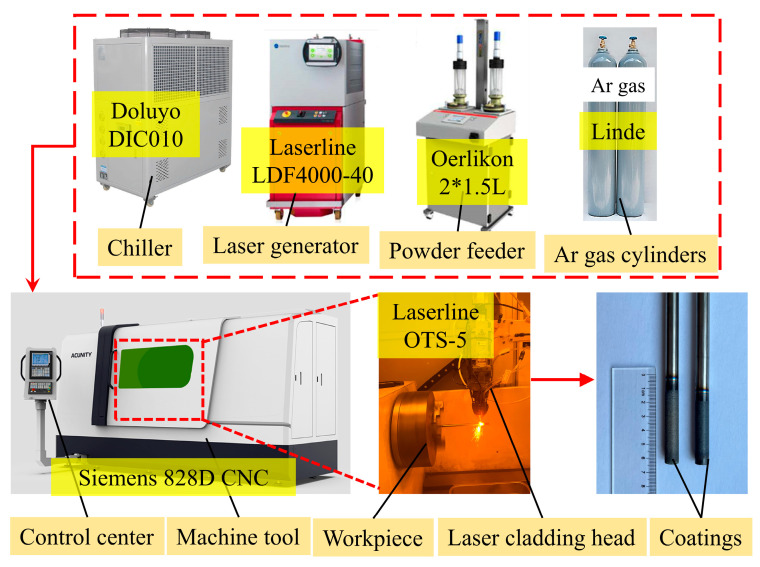
Coating preparation process.

The extreme high-speed laser cladding process was used to deposit a coating on the outer wall of Zr-4 alloy tubing. A Cr layer approximately 20-μm-thick was first deposited, followed by a 20-μm-thick FeCrAl (with Y_2_O_3_) layer. Figure 2 illustrates the coating preparation process and its deposition on the Zr-4 alloy. The optimized process parameters used for the deposition of the coating are provided in Table 3.

### 2.3. Oxidation Tests

A high-temperature isothermal steam testing system was employed to simulate reactor LOCA conditions. This system primarily consists of a steam generator (STA-HG, Suzhou Zhonghao Experimental Instrument Technology Co., Ltd., Suzhou, China), an Ar gas supply, and a horizontal tube furnace (BTF-1400C, Anhui BEQ Equipment Technology Co., Ltd., Hefei, China).

Before testing, both bare Zr-4 alloy tubes and coated Zr-4 alloy tubes were cut into 10-mm-long semi-cylindrical segments. The samples were placed in alumina crucibles and loaded into the furnace. The air inside the tube furnace was evacuated using a vacuum pump and then refilled with Ar gas to atmospheric pressure. The temperature was ramped to 1200 °C at a rate of 6 °C/min, with flowing argon gas (99.99% purity) introduced during heating to protect the samples from initial oxidation. Upon reaching 1200 °C, a uniform flow of steam (0.6 g/min) was introduced through the argon gas, and oxidation tests were conducted for durations of 10, 30, 60, and 120 min. The samples were then allowed to cool naturally to room temperature within the furnace. An analytical balance (accuracy:10^−4^ g) was used to weigh the samples prior to and following oxidation. The weight gain of the samples was calculated based on Equation (1):

(1) $$W=\frac{∆m}{A}$$

where *W* is the mass gain (mg/cm^2^), Δ*m* is the mass change of the sample before and after oxidation (mg), and *A* is the total surface area of the inner and outer surfaces and the two ends of the sample (cm^2^).

### 2.4. Microstructure and Composition Characterization

The phase composition of the samples before and after oxidation was analyzed using X-ray diffraction (XRD, Bruker Advance D8, Billerica, MA, USA) with Cu Kα radiation. The scan was performed with a step size of 0.02° over a 2θ range of 20° to 90°. The surface and cross-sectional morphology of the coatings before and after oxidation were observed using a scanning electron microscope (SEM, ZEISS GeminiSEM 300, Oberkochen, Germany). Microstructural element composition and distribution were analyzed by energy dispersive spectroscopy (EDS) at an acceleration voltage of 20 kV. The elastic modulus and hardness of the coating cross sections were tested using a nanoindenter (Bruker TI980) under a load of 4 mN. Five measurements were taken per sample, and the average value was calculated to minimize errors.

## 3. Results and Discussion

### 3.1. Microstructure of the Cladding Coatings

**Figure 3:**
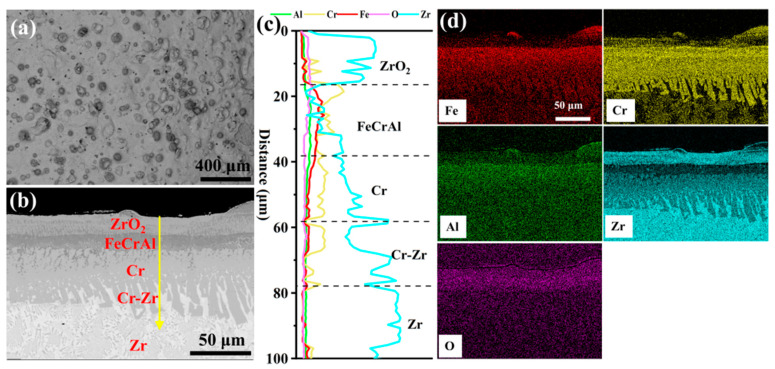
The morphologies of the cladding 0.0 wt.% Y_2_O_3_ coating: (**a**) Surface SEM image; (**b**) Cross-sectional SEM image; (**c**,**d**) Corresponding EDS scanning and mapping to (**b**).

Figure 3 depicts the surface and cross-sectional morphology of the 0.0 wt.% Y_2_O_3_ coating deposited on the Zr-4 alloy surface via EHLA. According to Figure 3a, the coating exhibits a defect-free surface morphology, devoid of macroscopic defects such as cracks or porosity. Spherical oxides of Fe, Cr, and Al are distributed across the surface, attributable to in situ oxidation of molten powder splats during rapid solidification (Figure S1). Additionally, thermocapillary convection driven by the Marangoni effect [18] facilitates Zr migration from the substrate to the coating surface, where it is oxidized, generating a continuous ZrO_2_ layer (~18-μm-thick). This oxide layer enhances the oxidation resistance of the coating [19]. The line scanning in Figure 3c indicates that an FeCrAl layer, approximately 20 μm thick, is deposited on a Cr layer of similar thickness (~20 μm). The Cr-Zr interface exhibits excellent metallurgical bonding, with the formation of a Cr-Zr intermetallic compound layer (~20-μm-thick).

**Figure 4:**
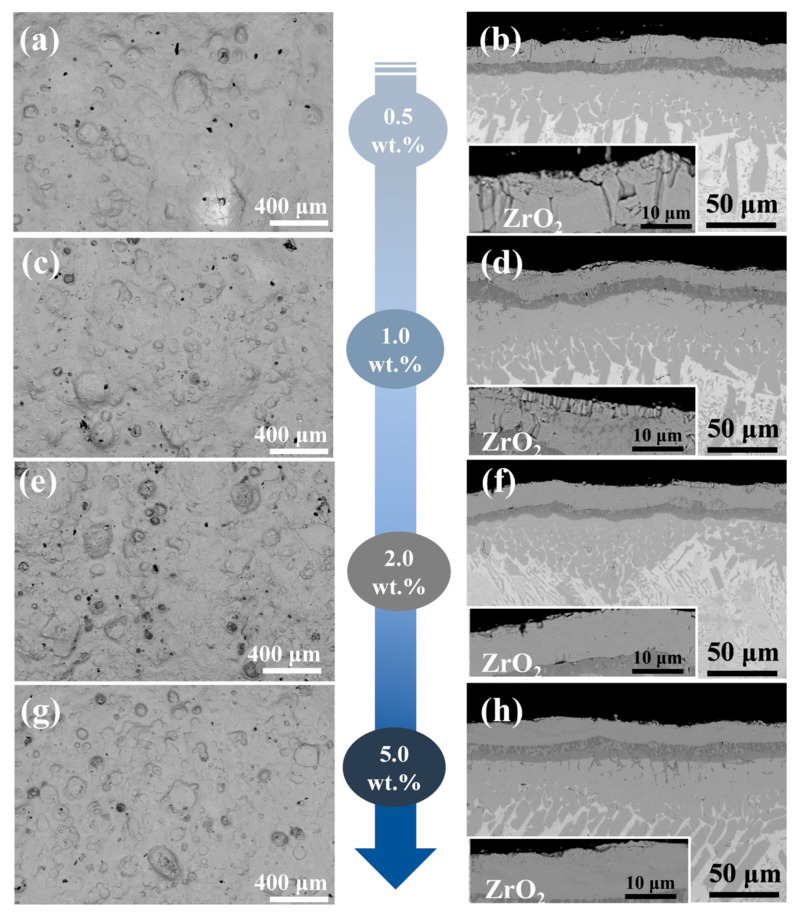
The surface and cross-sectional morphologies of the cladding Cr/FeCrAl-Y_2_O_3_ coating: (**a**,**b**) 0.5 wt.%; (**c**,**d**) 1.0 wt.%; (**e**,**f**) 2.0 wt.%; (**g**,**h**) 5.0 wt.%.

Figure 4a,c,e,g displays the surface morphology of four Y_2_O_3_-modified composite coatings (0.5–5.0 wt.% Y_2_O_3_), with corresponding EDS mappings shown in Figures S2–S5. The coating surfaces are dense, free of cracks or porosity. Compared to the Cr/FeCrAl coating, the addition of Y_2_O_3_ significantly reduces the presence of spherical oxides of Fe, Cr, and Al on the surface. This is likely due to the incorporation of Y, which effectively slows down the oxidation rate [20]. Furthermore, from Figures S2–S5, it can be observed that the distribution of Y is closely aligned with that of Zr. This is attributed to the similarity in atomic mass (Y: 88.906 amu; Zr: 91.224 amu), which leads to a tendency for Y atoms to cluster around Zr atoms. The cross-sectional morphology of the coatings is shown in Figure 4b,d,f,h, where the ZrO_2_ layer exhibits a columnar structure with a thickness ranging from 10–20 μm. The cross-sectional structure is divided into five layers from top to bottom: the ZrO_2_ layer, the FeCrAl coating, the Cr transition layer, the Cr-Zr bonding layer, and the Zr-4 substrate. The variation in coating thickness is closely related to the role of Y_2_O_3_ during laser cladding: An appropriate Y_2_O_3_ content (e.g., <5.0 wt.%) reduces thickness by increasing the molten pool’s latent heat (due to its high melting point), lowering the liquidus temperature, shortening solidification time, and suppressing elemental diffusion, thereby retaining more coating elements in the molten pool [21]. However, excessive Y_2_O_3_ (e.g., 5.0 wt.%) increases the dilution rate and thickness by hindering molten pool convection (due to its high melting point), reducing heat exchange efficiency, prolonging molten pool duration, and enhancing elemental diffusion.

**Figure 5:**
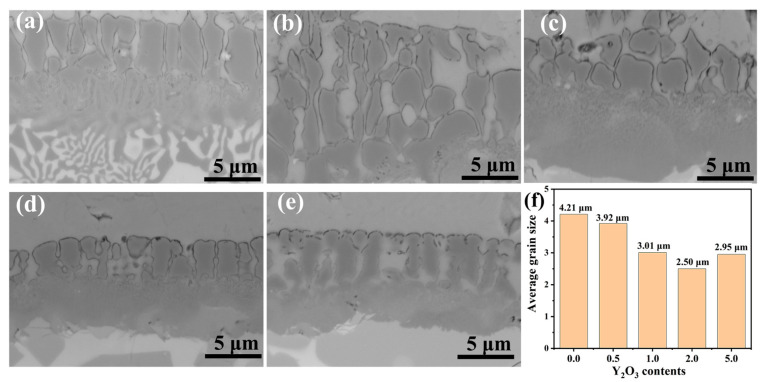
BSE image of grains in FeCrAl coatings: (**a**) 0.0 wt.%; (**b**) 0.5 wt.%; (**c**) 1.0 wt.%; (**d**) 2.0 wt.%; (**e**) 5.0 wt.%; (**f**) Average grain size of coatings.

Figure 5 depicts the microstructural evolution and average grain size in Y_2_O_3_-modified FeCrAl coatings. Due to the relatively large atomic radius of the rare earth element Y and its surface-active properties [22], Y atoms can reduce the surface tension of the nucleating crystals during solidification. This results in a decrease in the energy required to form critical-sized nuclei, thus promoting nucleation. Furthermore, Y atoms increase the activation energy for diffusion, which reduces the diffusion rate and slows down the growth of the grains, inhibiting grain coarsening. As quantified in Figure 5f, the coating containing 2.0 wt.% Y_2_O_3_ achieves smallest average grain size (~2.50 μm). This change may, to some extent, influence the oxidation characteristics of the coating.

**Figure 6:**
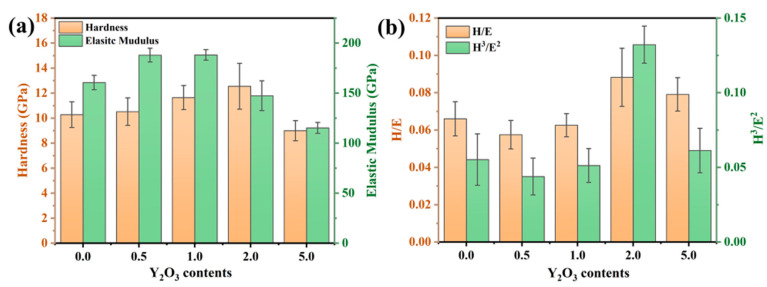
(**a**) Hardness and modulus of elasticity of FeCrAl coatings; (**b**) *H/E* and *H^3^/E^2^* ratio.

To investigate the effect of the rare-earth element Y on the mechanical properties of the coating, the hardness (H) and elastic modulus (E) of the FeCrAl coating were measured. As illustrated in Figure 6a, the nanoindentation hardness of the coating demonstrates a monotonic increase with Y_2_O_3_ concentration, achieving peak values (~12.5 GPa) at 2.0 wt.% Y_2_O_3_ doping. The addition of Y refines the grains by inhibiting grain growth. Consistent with the classical Hall–Petch relationship [23], the dispersion of secondary phase particles pinning the grain boundaries and dislocations impedes dislocation motion, thus enhancing the dispersion strengthening effect. Figure 6b presents the *H/E* and *H*^3^*/E*^2^ ratios of the coating. The *H/E* ratio is closely related to the coating’s resistance to damage and elastic strain capacity [24], while the *H*^3^*/E*^2^ ratio is indicative of the coating’s ability to resist crack initiation and propagation [25,26]. It can be observed that the *H*^3^*/E*^2^ ratio of the 2.0 wt.% coating is relatively high, indirectly confirming the refinement of the grains.

### 3.2. Oxidation Behavior

**Figure 7:**
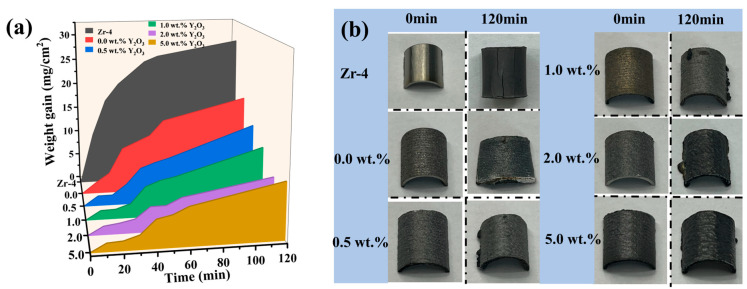
(**a**) Weight gain of Zr-4 and coatings in 1200 °C steam environment for different times; (**b**) Photographs of Zr-4 and coatings before and after 120 min oxidation.

Figure 7a illustrates the time-dependent curve of coating weight gain during exposure to 1200 °C steam. The results indicate that both the Zr-4 alloy and undoped Y_2_O_3_ coating (0.0 wt.%) experience a rapid increase in weight gain within the first 30 min, followed by a slower rate of weight gain. Conversely, the four Y_2_O_3_-doped coatings exhibit a slower weight gain in the initial 30 min, after which the oxidation rate significantly accelerates. After 120 min exposure, the Cr/FeCrAl coating without Y_2_O_3_ additive shows significantly lower mass gain (18.095 mg/ cm^2^) compared to Zr-4 alloy (28.517 mg/ cm^2^), representing a 37% reduction. Remarkably, the 2 wt.% Y_2_O_3_-modified coating achieves the lowest mass gain (8.749 mg/cm^2^), demonstrating optimal oxidation resistance among all specimens. Figure 7b compares the surface morphology of coatings pre- and post-oxidation. Severe cracking is observed on oxidized Zr-4 alloy, while the Y_2_O_3_-free coating displays thermal stress-induced plastic deformation. In contrast, Y_2_O_3_-containing coatings (0.5–2.0 wt.%) maintain structural integrity with minimal surface defects, suggesting enhanced thermal stability through rare-earth doping.

### 3.3. Phase Evolution

**Figure 8:**
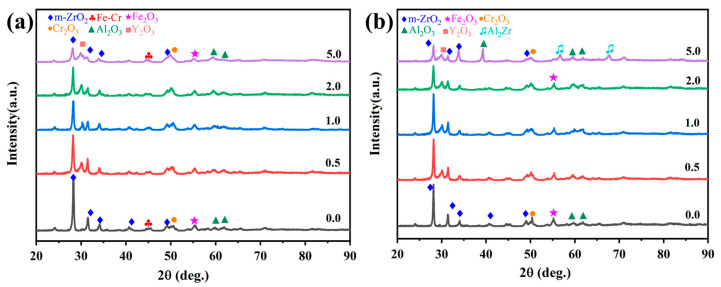
XRD patterns of the Cr/FeCrAl-based coatings before (**a**) and after 60 min oxidation (**b**).

Figure 8a,b comparatively presents the phase evolution of Cr/FeCrAl-based coatings through XRD analysis before and after 60-min oxidation at 1200 °C. As seen in Figure 8a, all five coatings exhibit characteristic peaks of m-ZrO_2_, Fe_2_O_3_, Cr_2_O_3_, and Al_2_O_3_ before oxidation. The m-ZrO_2_ layer has a higher crystallinity, which gives it a stronger scattering ability for X-rays. In contrast, the FeCrAl phase, with lower crystallinity and certain depth, typically exhibits lower peak intensity in the XRD pattern. XRD patterns of the FeCrAl powder and the as-polished FeCrAl coating are shown in Figure S6. Notably, Y_2_O_3_-doped coatings demonstrate prominent Y_2_O_3_ diffraction signals, where the m-ZrO_2_ peak intensity at 2θ = 28° progressively diminishes with increasing Y_2_O_3_ concentration. Figure 8b presents the post-oxidation XRD patterns, where the m- ZrO_2_ diffraction peak near 28° of the undoped coating shows a slight decrease in intensity compared to the pre-oxidation state. In comparison with the low-Y_2_O_3_ coatings, the 5.0 wt.% Y_2_O_3_ coating shows a lower intensity for the Cr_2_O_3_ peak and exhibits new characteristic peaks for Al_2_O_3_ and Al_2_Zr. This phase transformation primarily arises from the continuous oxidation of active Al elements and the competitive oxidation between Al/Zr elements [27].

### 3.4. Microstructure of the Coatings After Oxidation

**Figure 9:**
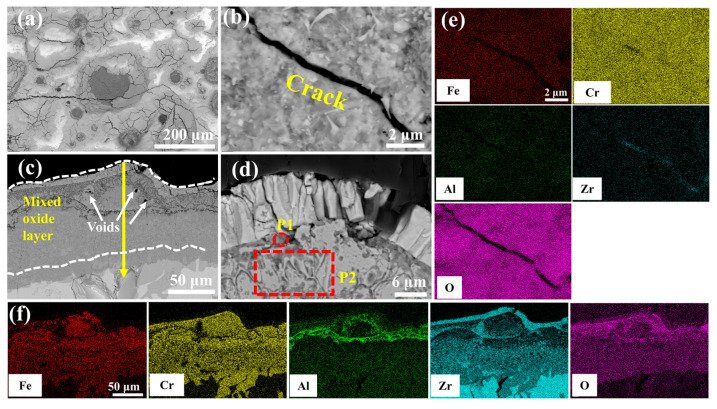
SEM morphologies of the 0.0 wt.% Y_2_O_3_ coating after 60 min oxidation: (**a**,**b**) Surface; (**c**,**d**) Cross-section; (**e**,**f**) EDS mappings of (**b**,**c**).

**Figure 10:**
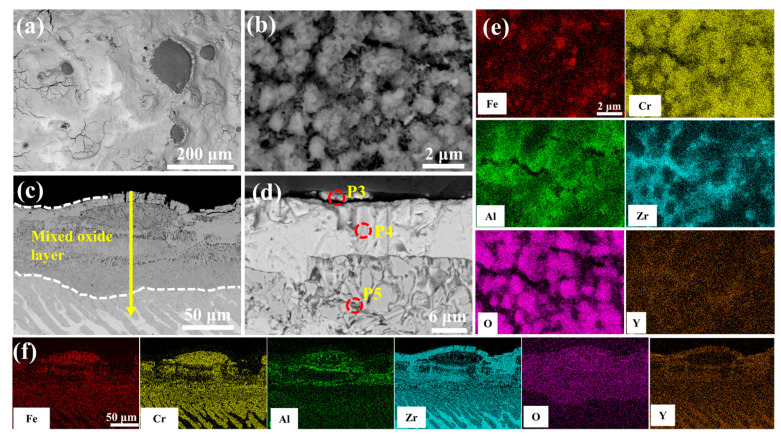
SEM morphologies of the 0.5 wt.% Y_2_O_3_ coating after 60 min oxidation: (**a**,**b**) Surface; (**c**,**d**) Cross-section; (**e**,**f**) EDS mappings of (**b**,**c**).

**Figure 11:**
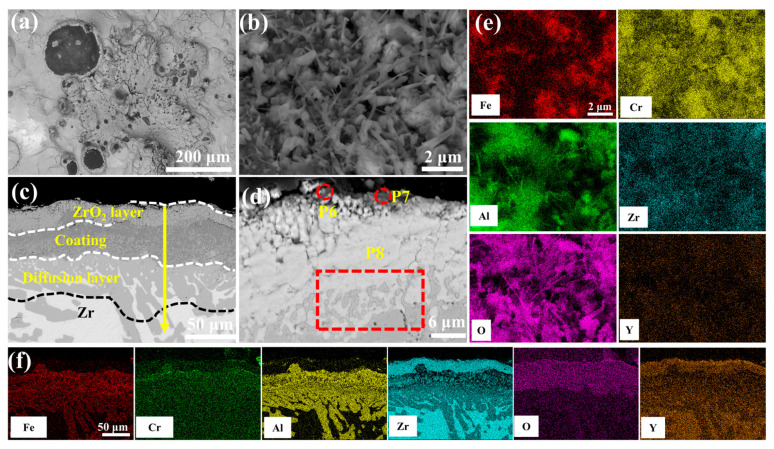
SEM morphologies of the 1.0 wt.% Y_2_O_3_ coating after 60 min oxidation: (**a**,**b**) Surface; (**c**,**d**) Cross-section; (**e**,**f**) EDS mappings of (**b**,**c**).

**Figure 12:**
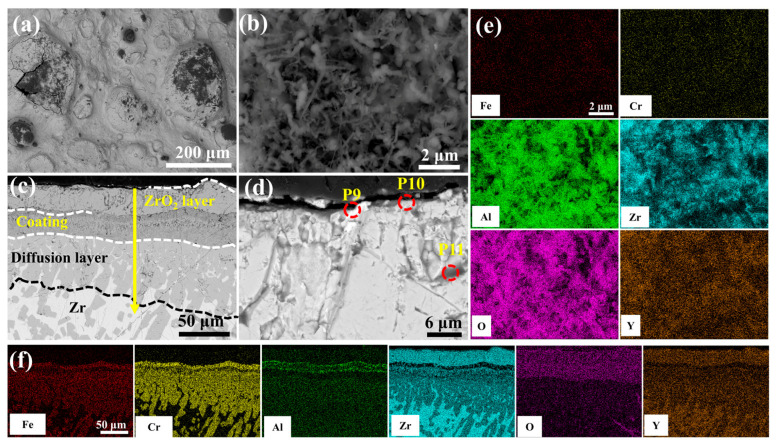
SEM morphologies of the 2.0 wt.% Y_2_O_3_ coating after 60 min oxidation: (**a**,**b**) Surface; (**c**,**d**) Cross-section; (**e**,**f**) EDS mappings of (**b**,**c**).

**Figure 13:**
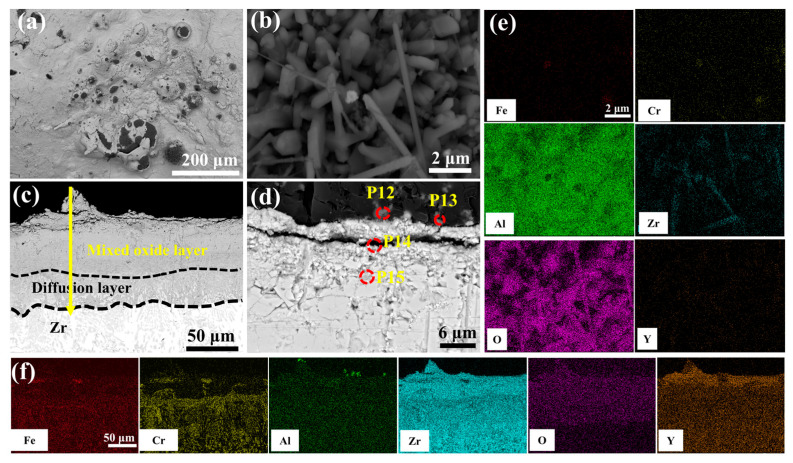
SEM morphologies of the 5.0 wt.% Y_2_O_3_ coating after 60 min oxidation: (**a**,**b**) Surface; (**c**,**d**) Cross-section; (**e**,**f**) EDS mappings of (**b**,**c**).

Figure 9 delineates the microstructural evolution of the undoped coating after 60 min of oxidation in a 1200 °C steam environment. Table 4 shows the elemental compositions of regions in Figure 9d, Figure 10d, Figure 11d, Figure 12d and Figure 13d. Irregular cracks are observed in the ZrO_2_ layer at protruding regions (Figure 9a). This observation aligns with Yang et al.’s report [28] demonstrating accelerated oxidation rate at convex surface features. The cracked ZrO_2_ layer exhibits large fissures (Figure 9b), exposing the underlying FeCrAl coating and accelerating further oxidation. Combined with the XRD results from Figure 8 and the EDS results from Figure 9e, it is evident that the main oxide phase in the coating is Cr_2_O_3_. The cross-sectional morphology of the coating (Figure 9c) shows that the cracks in the ZrO_2_ layer provide rapid diffusion pathways for oxygen, leading to the accelerated oxidation of Fe, Cr, and Al elements within the coating and the formation of large amounts of black, strip-like Al_2_O_3_. Furthermore, elemental diffusion results in the development of voids at the ZrO_2_ layer-coating interface.

Figure 10 characterizes the enhanced oxidation resistance of 0.5 wt.% Y_2_O_3_-modified coating through microstructural evolution in 1200 °C steam. As shown in Figure 10a, the number of surface cracks is significantly reduced compared to undoped counterparts. Discrete FeCrAl oxide clusters (diameter: 0.9–2.1 μm) are uniformly distributed across the stabilized ZrO_2_. The cross-sectional view in Figure 10c reveals that the EDS results at point 5 show lower concentrations of Fe, Cr, and Al compared to point 2, indicating a suppressed elemental interdiffusion. Notably, the Y_2_O_3_-doped system exhibits relatively obvious reduction of interfacial voids through inhibited Kirkendall effect. Moreover, the structure of the ZrO_2_ layer is denser (Figure 10d), in stark contrast to the coarse void structure observed in the Cr/FeCrAl coating in Figure 9d. This denser microstructure helps to prevent further oxygen penetration.

Figure 11 presents the morphological features of the 1.0 wt.% Y_2_O_3_ coating after 60 min of oxidation in a 1200 °C steam environment. As shown in Figure 11a, black oxides are observed on the surface. High-magnification imaging (Figure 11e) demonstrates the emergence of Al_2_O_3_ whiskers. These whiskers further restrict the diffusion of oxygen into the coating, thereby reducing the weight gain due to oxidation. Cross-sectional analysis (Figure 11c) quantifies oxygen penetration depth confined to 58.64 μm (vs. oxygen depth of 99.39 μm in Figure 10c). The oxide-coating interface at point 8 is well bonded with no voids, indicating that the coating exhibits enhanced resistance to oxidation at this stage.

Figure 12 delineates the optimal oxidation resistance of 2.0 wt.% Y_2_O_3_-modified coating in 1200 °C steam environment. Surface analysis (Figure 12b) reveals a continuous Al_2_O_3_ whisker-reinforced film with reduced Fe and Cr concentrations through selective oxidation. As seen in Figure 12c cross-sectional perspective, the depth of oxygen diffusion into the coating is minimal, and the oxidation weight gain is also the lowest. EDS scanning at point 11 confirms significant suppression of elemental interdiffusion in the ZrO_2_ layer. According to Figure S7, the coating can be divided into four structural layers: the mixed oxide layer, the residual coating, the Cr-Zr layer, and the Zr substrate. The mixed oxide layer consists of ZrO_2_, Fe_2_O_3_, Cr_2_O_3_, and Al_2_O_3_, with Cr diffusion into the substrate leading to an expansion of the Cr-Zr layer.

Figure 13 depicts the morphological evolution of the 5.0 wt.% Y_2_O_3_ coating following 60-min oxidation in 1200 °C steam. As illustrated in Figure 13b, the alumina whiskers evolve into needle-shaped structures, providing effective oxidation resistance. However, prolonged exposure induces microcracking within the initially dense ZrO_2_ layer (Figure 13d). This behavior is attributed to localized Y_2_O_3_ enrichment at the ZrO_2_ interface, which facilitates defect formation. The resultant interfacial cracks propagate preferentially along these defects, enhancing oxygen permeation and accelerating mass gain. Notably, the Cr element is almost undetectable on the surface (Figure 13e).

**Figure 14:**
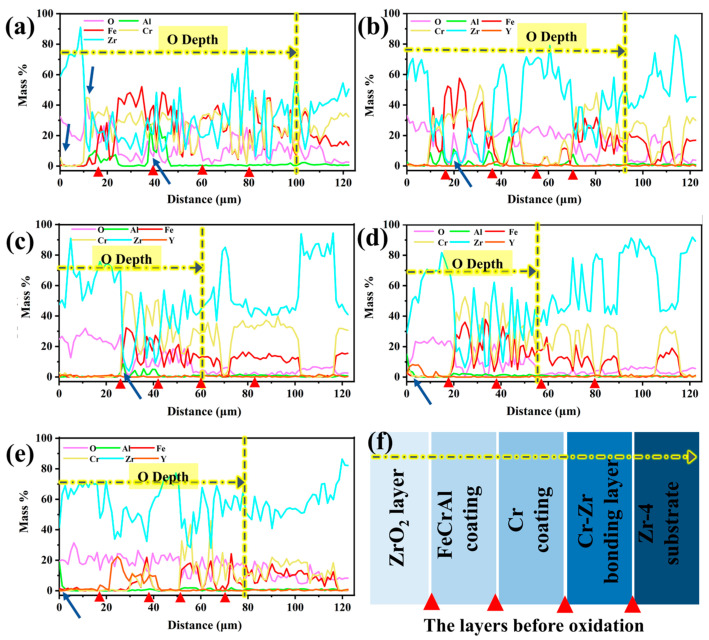
Depth-dependent elemental mass fraction distributions of the Y_2_O_3_-modified coatings after 60 min oxidation: (**a**) 0.0 wt.%; (**b**) 0.5 wt.%; (**c**) 1.0 wt.%; (**d**) 2.0 wt.%; (**e**) 5.0 wt.%; (**f**) The layers before oxidation.

Figure 14 depicts the depth-dependent elemental mass fraction distributions in Y_2_O_3_-doped coatings following 60-min oxidation. The red triangular markers in the figures correspond to the interfaces of the first five layers of the structure before oxidation, from the surface to the substrate: the ZrO_2_ layer, the FeCrAl coating, the Cr transition layer, the Cr-Zr bonding layer, and the Zr-4 substrate. As shown in Figure 14a,b, the oxygen penetration depths for the undoped Y_2_O_3_ coating and the 0.5 wt.% doped coating reach approximately 100 μm and 90 μm, respectively, indicating that rapid oxidation promotes the infiltration of oxygen into the Zr-4 substrate. As the Y_2_O_3_ doping level increases, the oxygen penetration depth gradually decreases, reaching a minimum value (~56 μm) when the doping amount is 2.0 wt.%. Further analysis reveals a significant element interdiffusion phenomenon in the undoped Y_2_O_3_ coating. Chromium and oxygen accumulate at the ZrO_2_ surface and interface (Figure 14a), which results from its upward diffusion and subsequent oxidation reaction. Simultaneously, the oxygen penetration induces intense oxidation of aluminum in the coating, as evidenced by the black-striped aluminum oxide in Figure 9c. As the Y_2_O_3_ doping level increases, the aluminum element shows a tendency to migrate towards the surface (indicated by arrows in Figure 14b–e). Notably, under low doping concentrations (0.0–0.5 wt.%), Fe and Cr elements migrate towards the surface and diffuse into the ZrO_2_ layer, whereas coatings with higher doping concentrations (1.0–5.0 wt.%) maintain interface stability. In particular, the 2.0 wt.% doped coating forms a continuous Al_2_O_3_ oxide film on the surface, with a higher mass fraction of Fe and Cr and lower oxygen content (average 9.6 wt.%) in the coating, suggesting that the diffusion in this coating is minimal and oxidation is slight, thus exhibiting optimal stability. When the doping level is increased to 5.0 wt.%, although the interface remains stable, the increased oxygen content leads to significant weight gain due to oxidation.

### 3.5. High-Temperature Steam Oxidation Mechanism

The high-temperature oxidation of FeCrAl coatings is fundamentally governed by the counter-diffusion of metal cations and oxygen anions through the oxide scale, followed by their reactive interaction at contact interfaces. The thermodynamic propensity of oxidation reactions was quantified through Gibbs free energy calculations performed in HSC Chemistry software (version 9.5.1), which yielded Equations (2)–(5). These equations establish that more negative values correlate with enhanced reaction spontaneity. The high affinity of Al and Cr in FeCrAl coatings toward water vapor promotes the surface formation of Cr_2_O_3_ and Al_2_O_3_, primarily driven by their outward diffusion and subsequent reaction with steam. Concurrently, thermal growth oxides induce localized expansion (Figure 9d, Table 4 P1), generating thermal stresses within the ZrO_2_ layer [29] that propagate cracks and trigger spallation, as evidenced by the attenuated m-ZrO_2_ diffraction peak intensity at 28° for the 0.0 wt.% Y_2_O_3_ coating in Figure 8b. Progressive oxidation intensifies elemental migration kinetics, generating interfacial voids at the ZrO_2_ boundary (Figure 9c and Figure S9a), which compromises interfacial adhesion through vacancy coalescence.

Post-oxidation surface oxides exhibit Y-dependent morphological and component evolution across all coatings, as systematically compared in Table S1 and Figure S8. Rare-earth doping effectively suppresses cation diffusion through Cr_2_O_3_ [30] and Al_2_O_3_ [31] scales while preserving anion transport pathways, as substantiated by prior studies. The Cr_2_O_3_ scale growth dominantly follows Cr^3+^ outward diffusion [32], leading to spherical black-gray Cr_2_O_3_ formation at low Y_2_O_3_ contents (0.0–1.0 wt.%), as shown in Figure 9b, Figure 10b, and Figure 11b. Enhanced Y_2_O_3_ addition facilitates Y segregation at grain boundaries, which impedes Cr^3+^ outward diffusion while promoting O^2-^ inward transport [33,34]. Given the inherently lower anionic versus cationic diffusivity in Cr_2_O_3_ [35], this dual effect substantially decelerates scale growth kinetics. In contrast, Al_2_O_3_ scale growth proceeds via O^2-^-dominant inward diffusion [36], with Al oxidation occurring preferentially within the coating matrix, as demonstrated in Figure 9c, Figure 10c and Figure 14a. Y incorporation effectively retards Al_2_O_3_ growth rates without altering its inward growth orientation, as consistently observed in Figure 11c, Figure 12c and Figure 13c. This Y-mediated cation diffusion suppression mitigates Kirkendall vacancy generation, thereby inhibiting void nucleation and minimizing interfacial porosity.

**Figure 15:**
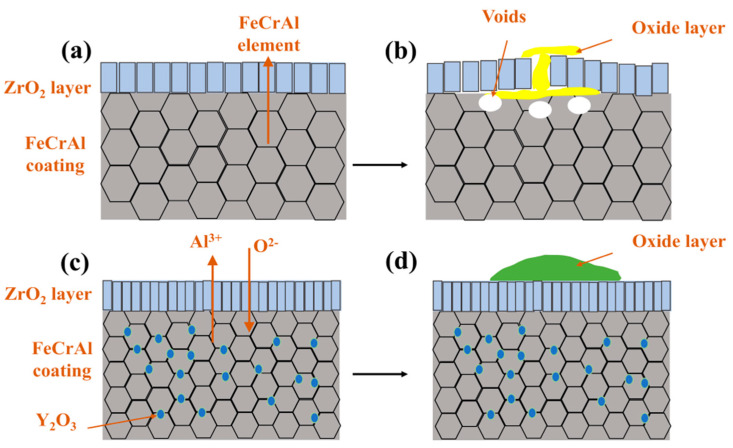
Oxidation mechanisms of FeCrAl coatings: (**a**,**b**) Without Y_2_O_3_; (**c**,**d**) With Y_2_O_3_.

The enhanced oxidation resistance of the 2.0 wt.% Y_2_O_3_-doped coating originates from Y-mediated regulation of oxide growth mechanisms: Y segregation at grain boundaries suppresses Cr^3+^ outward diffusion while promoting O^2-^ inward migration, thereby transforming the oxide growth mode from Cr^3+^-dominated epitaxial expansion to O^2-^-involved cooperative reactions. Concurrently, Y facilitates short-circuit diffusion of Al^3+^ along high-density grain boundary networks, accelerating the dynamic formation of a surface Al_2_O_3_ scale. This oxide scale, enriched with Y at grain boundaries, minimizes structural defects and forms a dense barrier against oxygen penetration (Figure 12b,e). Furthermore, the pinning effect of Y_2_O_3_ particles at ZrO_2_ grain boundaries effectively inhibited grain growth and promoted grain refinement. This microstructural evolution resulted in the formation of a continuous and homogeneous dense ZrO_2_ structure in both 2.0 and 5.0 wt.% Y_2_O_3_-doped samples (enlarged view of ZrO_2_ in Figure 4f,h). The structural characteristics were corroborated by X-ray diffraction analysis, as the diffraction peak intensity exhibits a direct correlation with atomic ordering within the crystal lattice. Generally, higher crystallinity corresponds to sharper characteristic diffraction peaks due to enhanced long-range atomic ordering [37]. Notably, the 5.0 wt.% Y_2_O_3_-doped coating demonstrated relatively lower ZrO_2_ diffraction peak intensities (Figure 8a), indicative of substantially refined crystallites. This refined structure enhances oxygen-blocking efficiency and thermal stress resistance by mitigating interfacial defects and optimizing thermal expansion matching. The grain boundary pinning effect of Y not only refines ZrO_2_ grains but also establishes dynamic equilibrium between suppressed cation diffusion (Cr^3+^) and regulated anion transport (O^2−^), synergistically optimizing elemental migration resistance, scale densification, and interfacial stability. Under these mechanisms, the 2.0 wt.% Y_2_O_3_ coating achieves a 52% reduction in oxidation-induced mass gain (8.749 mg/cm^2^) compared to undoped systems, demonstrating optimal high-temperature protective performance. Figure 15 depicts the oxidation mechanisms of FeCrAl coatings.

Excessive Y doping (>2.0 wt.%), however, induces ZrO_2_ embrittlement (Figure 13d) and accelerates inward oxygen diffusion, resulting in deleterious internal oxidation layer thickening (Figure 13c) that paradoxically increases mass gain and degrades coating durability.

(2) $$\frac{1}{2}Zr+H_{2}O\left(g\right)=\frac{1}{2}ZrO_{2}+H_{2}\left(g\;\right)\;\;(∆G=-244.9\;kJ/mol)$$

(3) $$\frac{2}{3}Al+H_{2}O\left(g\right)=\frac{1}{3}{Al}_{2}O_{3}+H_{2}\left(g\;\right)\;\;(∆G=-235.6\;kJ/mol)$$

(4) $$\frac{2}{3}Cr+H_{2}O\left(g\right)=\frac{1}{3}{Cr}_{2}O_{3}+H_{2}\left(g\;\right)\;\;(∆G=-86.7\;kJ/mol)$$

(5) $$\frac{2}{3}Fe+H_{2}O\left(g\right)=\frac{1}{3}{Fe}_{2}O_{3}+H_{2}\left(g\;\right)\;\;(∆G=18.3\;kJ/mol)$$

## 4. Conclusions

Cr/FeCrAl coatings with graded Y_2_O_3_ additions were fabricated on Zr-4 alloy via extreme high-speed laser cladding (EHLA) process, with a systematic investigation of the effects of Y_2_O_3_ addition on microstructural evolution and oxidation behavior in 1200 °C steam environments. Results revealed that Y doping effectively suppressed grain growth, inducing significant grain refinement in the FeCrAl coating and thereby enhancing coating hardness. Y enhanced Al diffusion through the coating, facilitating the formation of a protective Al_2_O_3_ scale. Furthermore, Y altered mass transport mechanisms during oxide growth by impeding outward cationic (Cr^3+^) diffusion along grain boundaries. This inhibition effectively restricts pore nucleation at the oxide-coating interface, thereby enhancing both scale adhesion and interfacial stability. The 2.0 wt.% Y_2_O_3_-modified coating exhibited optimal oxidation resistance, with the lowest mass gain (31% of Zr-4 alloy’s value) after 120-min steam exposure. The rare-earth oxide-doped composite coating fabricated via EHLA process in this study demonstrates viable applications in surface reinforcement engineering of Zr-4 alloy cladding tubes for nuclear reactors. This development provides practical guidance for developing accident-tolerant protective coatings against loss-of-coolant accidents (LOCA).

## Figures and Tables

**Table 1:** Chemical composition of Zr-4 alloy tubing (wt.%).

Element	Fe	Cr	Sn	C	Zr
Content	0.18–0.24	0.07–0.13	1.20–1.70	0–0.027	Bal.

**Table 2:** Elemental composition of coatings with different Y_2_O_3_ contents.

Sample	Composition of Coatings (wt.%)				Thickness (μm)
	Y_2_O_3_	Fe	Cr	Al	
#A	0.0	68.00	25.00	7.00	40
#B	0.5	67.66	24.875	6.965	39
#C	1.0	67.32	24.75	6.93	38
#D	2.0	66.64	24.5	6.86	32
#E	5.0	64.6	23.75	6.65	35

**Table 3:** Optimized process parameters for depositing coating.

Parameter	Laser Power (kW)		Cladding Speed (m/min)	Track Overlap (mm)	Powder Flow Rate (g/min)
Value	0.4 (Cr)	0.45 (FeCrAl-Y_2_O_3_)	20	0.6	3.91

**Table 4:** Elemental compositions (EDS) of regions in Figure 9d, Figure 10d, Figure 11d, Figure 12d and Figure 13d (wt.%).

Composition (wt.%)	Fe	Cr	Al	Y	Zr	O
P1	1.45	61.61	4.44	-	9.14	23.36
P2	22.84	32.34	5.58	-	17.49	21.74
P3	1.10	1.47	0.14	-	86.05	11.24
P4	0.63	1.09	0.06	-	69.67	28.55
P5	14.55	21.27	9.79	-	30.01	24.37
P6	0.73	2.42	0.49	12.17	57.45	26.75
P7	1.62	1.45	4.01	20.37	43.27	29.29
P8	12.81	20.28	1.72	-	48.60	16.59
P9	8.63	0.71	3.40	0.27	61.26	25.73
P10	3.23	1.85	0.10	2.74	49.75	42.35
P11	0.08	0.21	-	-	47.76	51.95
P12	0.26	0.10	46.46	1.44	8.33	43.40
P13	0.21	0.47	31.39	4.30	15.83	47.81
P14	0.35	-	-	10.73	65.15	23.77
P15	0.25	0.06	0.18	10.45	65.20	23.86

## Data Availability

The original contributions presented in the study are included in the article/Supplementary Material, further inquiries can be directed to the corresponding authors.

## Supplementary Materials

The following supporting information can be downloaded at: https://www.mdpi.com/article/10.3390/ma18081821/s1. Figures S1–S5: The EDS mappings of the Y_2_O_3_-modified coatings before oxidation. Figure S6: XRD patterns of the FeCrAl powder and the as-polished FeCrAl coating. Figure S7: Cross-sectional morphology of 2.0 wt.% Y_2_O_3_ coating after 60 min oxidation. Table S1: Quantitative results of EDS mappings elements in Figure 9e, Figure 10e, Figure 11e, Figure 12e and Figure 13e. Figures S8 and S9: Evolution of surface and cross-sectional morphologies of the Y_2_O_3_-modified coatings after oxidation.
