# Supplementary material for: Effect of Yttrium Oxide on Microstructure and Oxidation Behavior of Cr/FeCrAl Coatings Fabricated by Extreme High-Speed Laser Cladding Process: An Experimental Approach
Source: Materials (Basel). 2025 Apr 16;18(8):1821. doi: 10.3390/ma18081821 (PMC12028746; doi:10.3390/ma18081821)
Supplement: Supplementary file 1 [file materials-18-01821-s001.zip › materials-3540126-supplementary.pdf]

Supporting Information

Effect of Yttrium Oxide on Microstructure and Oxidation Behavior of Cr/FeCrAl Coatings Fabricated by Extreme High-Speed Laser Cladding Process: An Experimental Approach

Tian Liang [1,2], Jian Liu [2], Chi Zhan [3], Shaoyuan Peng [3,*] and Jibin Pu [2,*]

[1] School of Materials Science and Chemical Engineering, Ningbo University, Ningbo 315211, China; liangtian@nimte.ac.cn

[2] State Key Laboratory of Advanced Marine Materials, Ningbo Institute of Materials Technology and Engineering, Chinese Academy of Sciences, Ningbo 315201, China; liujian123@nimte.ac.cn

[3] China Merchants Marine and Offshore Research Institute Co., Ltd., Shenzhen 518066, China; zhanchi1@cmhk.com

* Correspondence: pengshaoyuan1@cmhk.com (S.P.); pujibin@nimte.ac.cn (J.P.)

Figures S1-S5: The EDS mappings of the Y_2O_3-modified coatings before oxidation.

Figure S6: XRD patterns of the FeCrAl powder and the as-polished FeCrAl coating.

Figure S7: Cross-sectional morphology of 2.0 wt.% Y_2O_3 coating after 60 min oxidation.

Table S1: Quantitative results of EDS mappings elements in Figures 9e-13e.

Figures S8-S9: Evolution of surface and cross-sectional morphologies of the Y_2O_3-modified coatings after oxidation.

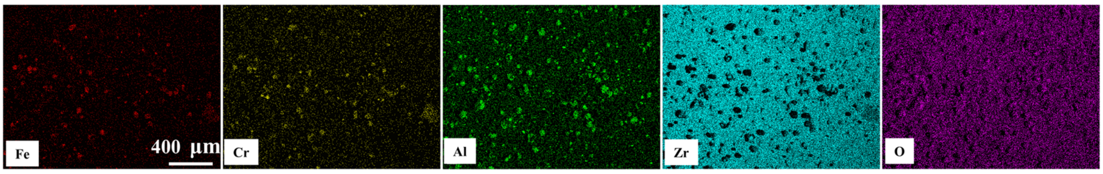

Figure S1. The corresponding EDS mapping to Figure 3a.

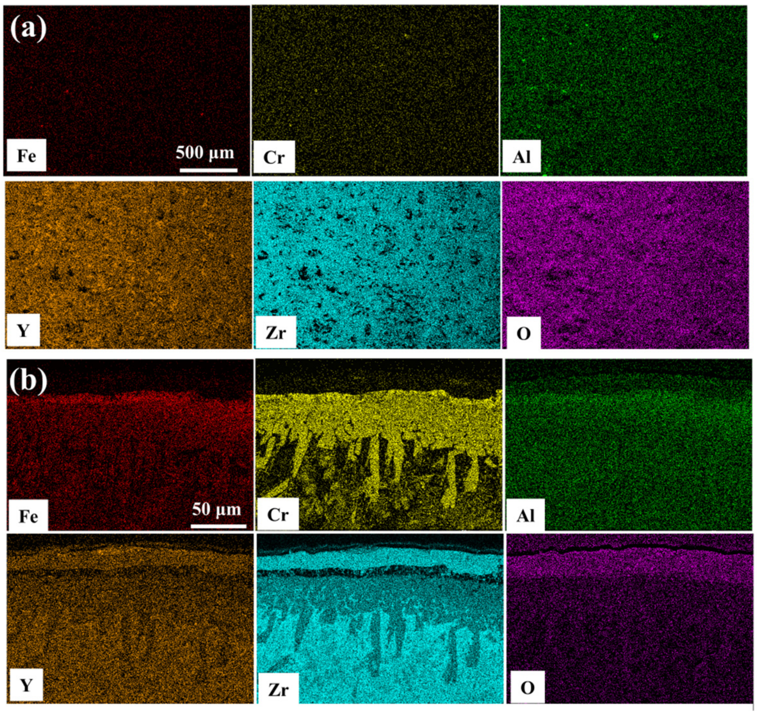

Figure S2. EDS mappings of 0.5 wt.% Y_2O_3 coating: (a) surface; (b) cross-section.

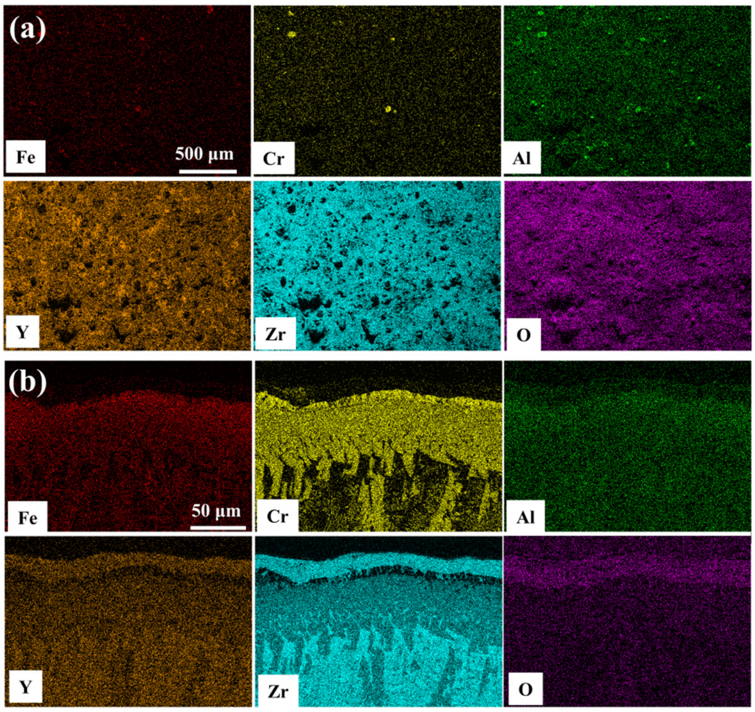

Figure S3. EDS mappings of 1.0 wt.% Y_2O_3 coating: (a) surface; (b) cross-section.

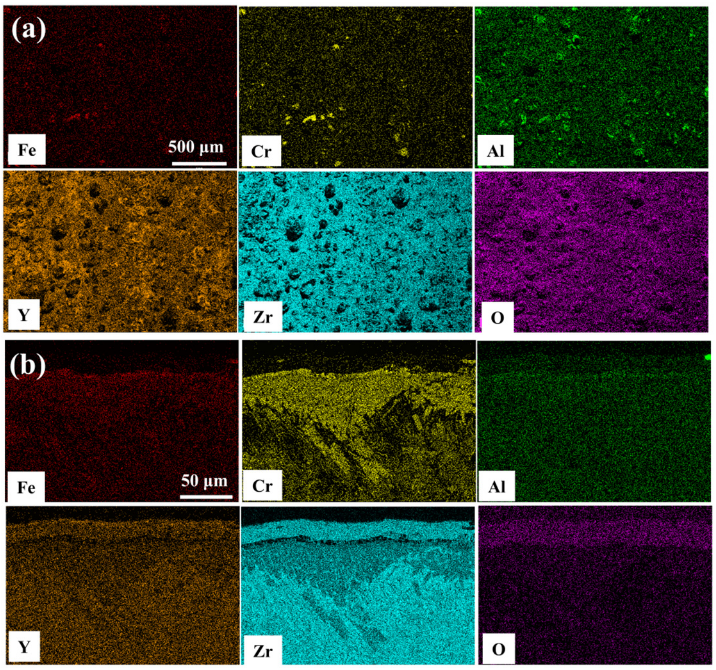

Figure S4. EDS mappings of 2.0 wt.% Y_2O_3 coating: (a) surface; (b) cross-section.

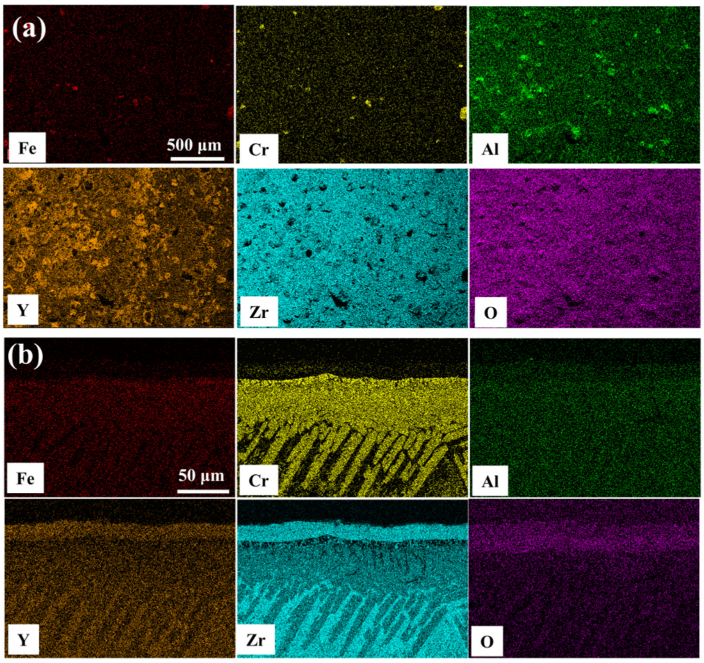

Figure S5. EDS mappings of 5.0 wt.% Y_2O_3 coating: (a) surface; (b) cross-section.

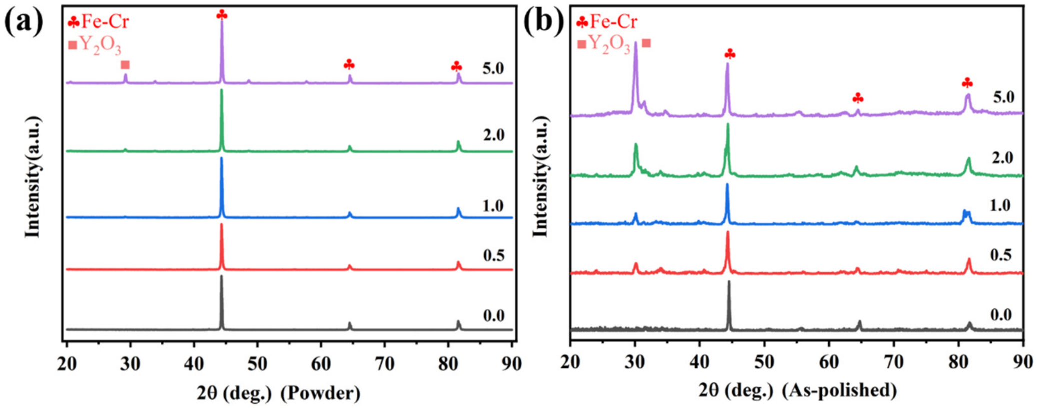

Figure S6. XRD patterns of the FeCrAl powder and the as-polished FeCrAl coating.

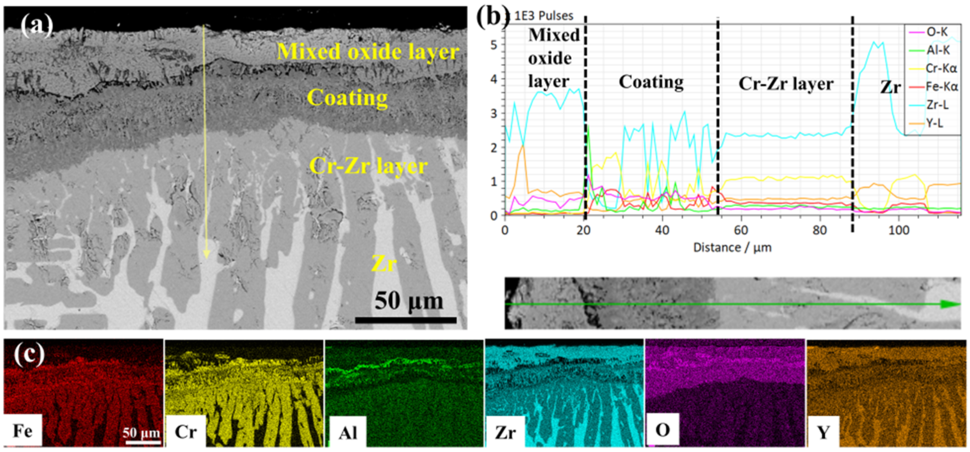

Figure S7. Cross-sectional morphology of 2.0 wt.% Y_2O_3 coating after 60 min oxidation: (a) SEM image; (b, c) EDS scanning and mapping.

Table S1 Quantitative results of EDS mapping elements in Figures 9e-13e.

Sample (wt.%)	Fe	Cr	Al	Y	Zr	O
0.0	13.75	49.73	0.27	-	0.44	35.82
0.5	4.43	55.20	1.43	-	1.46	37.47
1.0	6.38	24.98	28.88	0.01	5.65	34.10
2.0	0.36	0.31	45.38	-	7.13	48.62
5.0	1.36	1.56	46.42	0.09	1.30	49.26

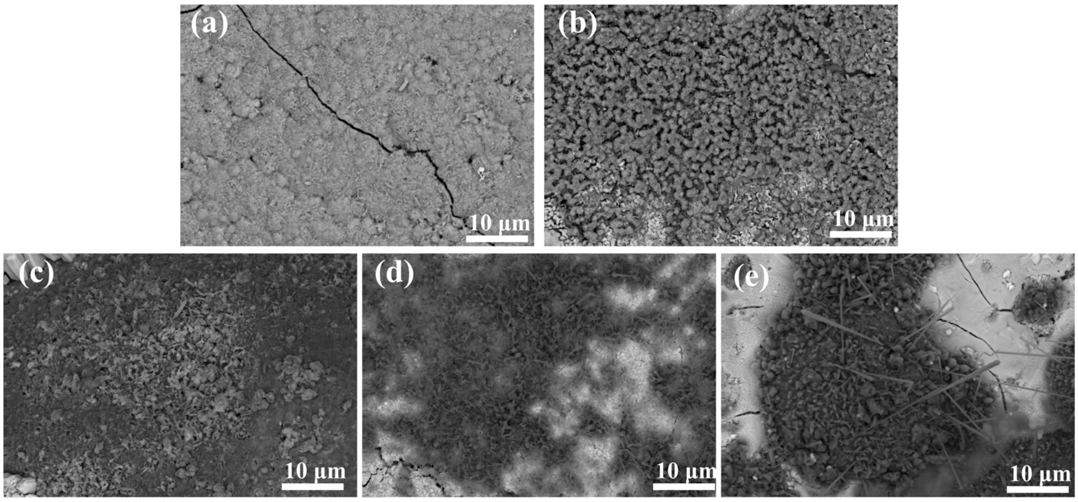

Figure S8. Evolution of surface morphologies of the Y_2O_3-modified coatings after 60 min oxidation: (a) 0.0 wt.%; (b) 0.5 wt.%; (c) 1.0 wt.%; (d) 2.0 wt.%; (e) 5.0 wt.%.

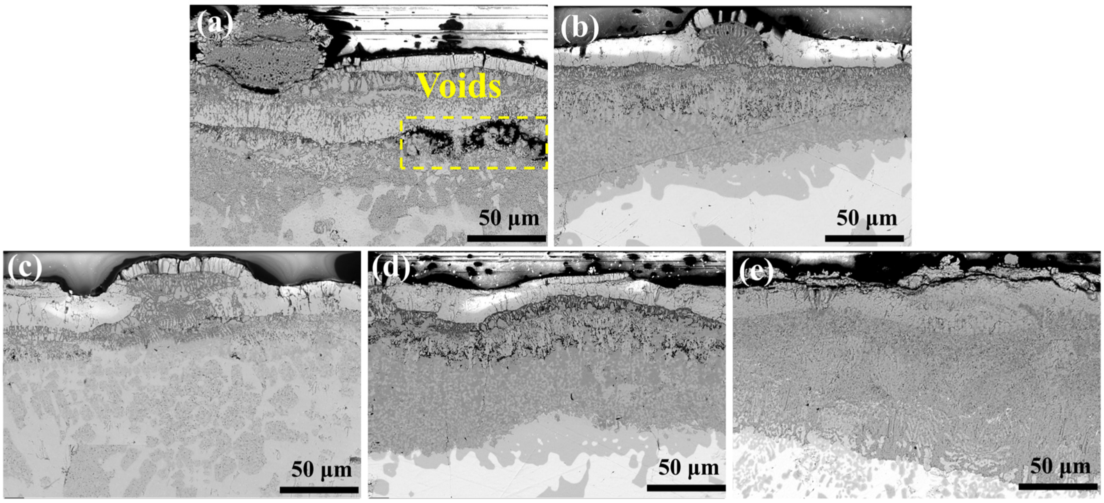

Figure S9. Evolution of cross-sectional morphologies of the Y_2O_3-modified coatings after 120 min oxidation: (a) 0.0 wt.%; (b) 0.5 wt.%; (c) 1.0 wt.%; (d) 2.0 wt.%; (e) 5.0 wt.%.
